# Racial disparities in initiation of chemotherapy among breast cancer patients with discretionary treatment indication in the state of Georgia

**DOI:** 10.1007/s10549-024-07279-w

**Published:** 2024-03-22

**Authors:** Lindsay J. Collin, Jade Jones, Rebecca Nash, Jeffrey M. Switchenko, Kevin C. Ward, Lauren E. McCullough

**Affiliations:** 1 Department of Population Health Sciences, Huntsman Cancer Institute, University of Utah, 2000 Circle of Hope Dr, Salt Lake City, UT 84112 USA; 2 Department of Hematology and Medical Oncology, Emory University School of Medicine, Atlanta, USA; 3 Department of Epidemiology, Rollins School of Public Health, Emory University, Atlanta, GA USA; 4 Department of Biostatistics, Rollins School of Public Health, Emory University, Atlanta, GA USA

**Keywords:** Breast cancer, Cancer health disparities, Survivorship, Chemotherapy

## Abstract

**Purpose:**

The majority of breast cancer patients are diagnosed with early-stage estrogen receptor (ER) positive disease. Despite effective treatments for these cancers, Black women have higher mortality than White women. We investigated demographic and clinical factors associated with receipt of chemotherapy among those with a discretionary indication who are at risk for overtreatment.

**Methods:**

Using Georgia Cancer Registry data, we identified females diagnosed with ER positive breast cancer who had a discretionary indication for chemotherapy (2010–2017). We used logistic regression to estimate odds ratios (ORs) and 95% confidence intervals (CIs) associating patient demographic and clinical characteristics with chemotherapy initiation overall, and comparing non-Hispanic Black (NHB) with non-Hispanic White (NHW) women within strata of patient factors.

**Results:**

We identified 11,993 ER positive breast cancer patients with a discretionary indication for chemotherapy. NHB patients were more likely to initiate chemotherapy compared with NHW women (OR = 1.41, 95% CI: 1.28, 1.56). Race differences in chemotherapy initiation were pronounced among those who did not receive Oncotype DX testing (OR = 1.47, 95% CI: 1.31, 1.65) and among those residing in high socioeconomic status neighborhoods (OR = 2.48, 95% CI: 1.70, 3.61). However, we observed equitable chemotherapy receipt among patients who received Oncotype DX testing (OR = 0.90, 95% CI: 0.71, 1.14), were diagnosed with grade 1 disease (OR = 1.00, 95% CI: 0.74, 1.37), and those resided in rural areas (OR = 1.01, 95% CI: 0.76, 1.36).

**Conclusion:**

We observed racial disparities in the initiation of chemotherapy overall and by sociodemographic and clinical factors, and more equitable outcomes when clinical guidelines were followed.

**Supplementary Information:**

The online version contains supplementary material available at 10.1007/s10549-024-07279-w.

## Introduction

Breast cancer is the most commonly diagnosed cancer among women in the United States (US) and the second leading cause of cancer-specific mortality despite a high five-year relative survival of approximately 90% [1–3]. The majority of breast cancer patients present with early-stage (91%), estrogen receptor (ER) positive disease (~ 75%), and/or no lymph node involvement (~ 65%) at diagnosis, which also contribute to the overall favorable prognosis [3–5]. Among breast cancer patients diagnosed with ER-positive disease and no lymph node involvement, chemotherapy may be considered a discretionary treatment according to the National Comprehensive Cancer Network (NCCN) clinical guidelines [6]. Chemotherapy can have severe side effects that impact quality of life [6], including neuropathy, cognitive impairment (*i.e.,* “chemo brain”), neutropenia, infections, and other poor patient-reported outcomes. Therefore, ensuring that unnecessary chemotherapy is avoided is critical to improving quality of life for breast cancer survivors.

Black women in the US experience persistent disparities in breast cancer mortality compared with White women [7–10]. Notably, these racial disparities in mortality are most pronounced for breast cancers with effective treatment regimens [11]. Our group has previously reported that Black and White women in the metropolitan Atlanta area diagnosed with nonmetastatic breast cancer were equally likely to receive guideline-concordant care [5]. However, Black women were more likely to receive chemotherapy than White women, with the most notable discrepancies in receipt of chemotherapy among patients with a discretionary indication for chemotherapy [5]. Previous studies have suggested that Black women are more likely to receive chemotherapy than other racial and ethnic groups, which may lead to overtreatment among Black women expected to have a favorable prognosis [12, 13]. In instances when chemotherapy is considered discretionary, Black women may be more likely to receive therapy due to the concern that their outcomes (*i.e.,* recurrence or mortality) are often worse than other racial and ethnic groups. However, the decision-making process on care protocols must also weigh the concerns of overtreatment due to the negative side effects of chemotherapy [10]. A previous study of breast cancer patients reported that receipt of chemotherapy among women with a discretionary indication for chemotherapy was associated with clinical predictors of poor outcomes [14]. This study did not find differences in the initiation of chemotherapy by race, but was limited in the approach due to a small number of Black women (*n* = 61) and it did not examine differences in chemotherapy initiation across patient characteristics [14].

We sought to understand patient demographic and clinical factors associated with the receipt of chemotherapy among women with a discretionary indication for chemotherapy who may be at risk for overtreatment. We examined potential race differences in chemotherapy initiation among non-Hispanic Black (NHB) and non-Hispanic White (NHW) women overall and across strata of important demographic and clinical factors.

## Methods

### Study population

Using the Georgia Cancer Registry (GCR), we identified women diagnosed with breast cancer between 2010 and 2017 while residing in the state of Georgia. Breast cancer patients were included if they were diagnosed with an invasive first primary ER-positive breast tumor and were classified as being NHB or NHW [15]. Race is a social construct, representing the social and structural interactions to maintain the privilege, power, and resource aggregation of the dominant group. Therefore, throughout this manuscript, we evaluated potential racial disparities in chemotherapy receipt among breast cancer patients. We identified ER positive breast cancer patients at risk for overtreatment, including women who were considered discretionary for chemotherapy according to the 2016 NCCN guidelines (Supplemental Table 1) [6, 16]. These included patients diagnosed with: (1) ER-positive/HER2-positive breast cancer with no lymph node involvement and tumor size ≤ 1.0 cm, and (2) ER-positive/HER2-negative breast cancer with no lymph node involvement and tumor size > 0.5 cm–5 cm, who received an Oncotype DX recurrence score of Intermediate (18–30), or (3) ER-positive/HER2-negative breast cancer with no lymph node involvement and tumor size > 0.5 cm–5 cm, who did not receive Oncotype DX testing.

In an additional analysis, we identified women for which chemotherapy is indicated, but they had limited lymph node involvement (1–3 positive nodes) [6, 16]. We included this analysis because we previously observed that NHB breast cancer patients were more likely to initiate chemotherapy than NHW patients in this subset of patients [5].

### Outcome assessment

The primary outcome of interest was chemotherapy initiation. Initiation of chemotherapy was available from the GCR and was coded as yes or no. The GCR collects information on first-line therapies received as well as their dates of initiation. We defined chemotherapy initiation as receipt of chemotherapy following breast cancer diagnosis in our study. However, systemic therapies are incompletely captured in cancer registries and “no” can both refer to not receiving chemotherapy or unknown receipt of chemotherapy. Our secondary outcome of interest was breast cancer-specific mortality. Breast cancer mortality (ICD10-C50) was determined from death certificate data. Follow-up was defined as time in months, from the date of diagnosis until the first of a) death, b) last date of contact in the registry, or c) December 31, 2019, the end of study follow-up.

### Patient demographic and clinical characteristics

We collected information on patient demographic and clinical characteristics that may influence initiation of chemotherapy. These clinical factors included age at diagnosis (years, continuous), tumor stage (I–III), tumor grade (1, 2, 3 +), tumor size (cm), number of positive lymph nodes (0, 1, 2, 3), and receipt of Oncotype DX testing (yes, no). We also collected demographic characteristics including race and ethnicity (NHB, NHW), marital status (single, married/domestic partner, divorced/separated/other), insurance status (private, Medicare, Medicaid, military/other, and uninsured), urban or rural residence, and a Census-derived area-based measure of socioeconomic status [SES] (0%– < 5%, 5%– < 10%, 10%– < 20%, 20%–100% below poverty). SES was based on Census tract-level poverty data published annually from the American Community Survey [17, 18].

### Statistical methods

Descriptive statistics were calculated as median values and interquartile range (IQR) or frequency and percent for covariates of interest across categories of chemotherapy initiation. We used multivariable-adjusted logistic regression models to compute the odds ratios (OR) and 95% confidence intervals (CI) associating patient demographic and clinical characteristics with the initiation of chemotherapy. We further computed racial disparities in receipt of chemotherapy, and whether clinical and patient demographic characteristics modified these associations by comparing stratum-specific estimates of association. We also explored the associations of clinical and patient demographic characteristics with initiation of chemotherapy by age (< 55 years, ≥ 55 years). We did this because women diagnosed with breast cancer at a younger age may be treated more aggressively than women diagnosed later in life and, therefore, may be more likely to receive chemotherapy. In an additional analysis, we examined associations of sociodemographic and clinical characteristics with chemotherapy initiation among breast cancer patients diagnosed with ER-positive disease and limited lymph node involvement (Supplemental Tables 2, 3, and 4).

We then examined racial disparities in breast cancer mortality within strata of chemotherapy initiation by using Cox proportional hazards regression to compute the hazard ratios (HR) and 95% CIs. Due to confounding by indication [19], we could not reliably compute overall estimates for the association between chemotherapy initiation and breast cancer mortality and therefore only present racial disparities in breast cancer mortality within stratum of chemotherapy initiation. The proportional hazards assumption was assessed by visual inspection of the ln-ln survival curves.

For all models, we assessed confounding based on a priori knowledge and graphical-based methods (DAGs) [20]. For the associations of patient demographic and clinical characteristics with chemotherapy initiation, potential confounders included age at diagnosis, tumor stage, tumor grade, SES, insurance status, and marital status. In the models examining racial disparities in breast cancer mortality, we present age-adjusted analyses for the racial disparity as well as multivariable-adjusted models, including age, stage, grade, insurance status, SES, receipt of Oncotype DX testing, marital status, and urban/rural geography. No hypothesis testing was performed [21, 22]. All analyses were conducted using SAS v9.4 (Cary, NC).

## Results

The study cohort included 11,993 breast cancer patients with ER-positive disease and a discretionary indication for chemotherapy of which 2,882 (24%) were NHB and 9,111 (76%) were NHW (Table 1). The distribution of discretionary groups from Table 1 were comparable across NHW and NHB patients (ER + /HER2 + : 5.7%, 6.9%; ER + /HER2 − and Oncotype DX Intermediate: 16%, 16%; ER + /HER2 − no Oncotype DX testing: 78%, 77%, respectively; data not shown). In Table 1 we compare the distribution of patient demographic and clinical characteristics among those who did and did not receive chemotherapy. In this cohort, 2,872 (24%) initiated chemotherapy. NHB women were more likely to initiate chemotherapy than NHW women (33% vs. 21%). Breast cancer patients who initiated chemotherapy were 55 years of age on average (IQR: 46, 63 years) compared with 66 years of age among those who did not initiate chemotherapy (IQR: 58, 74). Breast cancer patients who were single at the time of diagnosis were more likely to initiate chemotherapy than those who were married at the time of diagnosis (34% vs. 26%, respectively). As expected, patients diagnosed with more aggressive disease (*i.e.,* later stage, higher grade, and larger tumor size) were more likely to receive chemotherapy. Initiation of chemotherapy did not vary by socioeconomic index or urban/rural residency. Individuals who had Medicaid insurance were more likely to initiate chemotherapy compared with those who had Medicare insurance (42% vs. 11%).

**Table 1:** Demographic, tumor, and patient characteristics by receipt of chemotherapy among 11,993 non-Hispanic Black (NHB) and non-Hispanic White (NHW) women diagnosed with ER + breast cancer and discretionary indication for chemotherapy diagnosed in Georgia (2010–2017)

	Chemotherapy initiation			
	Yes		No	
	(*n* = 2,872)		(*n* = 9,121)	
	Median	IQR	Median	IQR
Age at diagnosis (years)	55	46, 63	66	58, 74
Length of follow-up (months)	67	44, 92	56	39, 84
	**N**	**%**	**N**	**%**
Breast cancer-specific death	165	5.8	21	2.4
Race and ethnicity				
NHB	942	33	1940	67
NHW	1930	21	7181	79
Age at diagnosis				
> 70	182	5.3	3268	95
55–70	1180	23	3997	77
≤ 55	1510	45	1856	55
Stage				
I	1717	19	7512	81
II	1155	42	1609	58
Tumor grade				
1	286	7.6	3498	92
2	1145	21	4380	79
3 +	1395	57	1061	43
Unknown	46	20	182	80
Tumor size (cm)				
≤ 0.5	76	26	222	74
0.6–1	615	16	3251	84
> 1 to < 5	2181	29	5648	72
Unknown				
ODX testing				
Not performed	2228	22	7794	78
Performed	644	33	1327	67
Subtype				
ER + /HER2-	2539	23	8744	77
ER + /HER2 +	333	47	377	53
Demographic characteristics				
Marital status				
Single	469	34	921	66
Married (common law and unmarried domestic)	1719	26	4826	74
Other (divorced, widowed, separated)	575	16	2957	84
Unknown	109	21	417	79
Socioeconomic Index				
0% – < 5% poverty	392	23	1340	77
5% – < 10% poverty	581	22	2017	78
10% – < 20% poverty	1037	25	3088	75
20–100% poverty	862	24	2676	76
Urban/rural residence				
Urban	2382	24	7374	76
Rural	489	22	1747	78
Insurance type				
Uninsured	40	33	82	67
Private	1896	34	3665	66
Medicaid	238	42	335	58
Medicare	584	11	4749	89
Military	71	32	151	68
Unknown	42	23	138	77

In Table 2 we present the multivariable-adjusted associations relating clinical and patient demographic characteristics with receipt of chemotherapy. Among all women who were discretionary for chemotherapy, breast cancer patients who were > 70 years at diagnosis had lower odds of receiving chemotherapy (OR = 0.72, 95% CI: 0.52, 0.98) than those who were ≤ 55 years at diagnosis. However, breast cancer patients who were 56–70 years at diagnosis had higher odds of receiving chemotherapy than those who were ≤ 55 years at diagnosis (OR = 1.28, 95% CI: 1.08, 1.51). As expected, increasing stage and grade were associated with an increased odds of receipt of chemotherapy. Interestingly, those who did not receive Oncotype DX testing had lower odds of receiving chemotherapy (OR = 0.77, 95% CI: 0.69, 0.87) than those who received an Oncotype DX test. Breast cancer patients residing in neighborhoods with low SES had 1.3-times to the odds of receiving chemotherapy than those who lived in high SES neighborhoods (OR = 1.35, 95% CI: 1.15, 1.58). We did not observe a difference in chemotherapy initiation by urbanicity (OR = 0.95, 95% CI: 0.83, 1.08). Breast cancer patients with Medicaid insurance had higher odds of receiving chemotherapy than those with private insurance (OR = 1.25, 95% CI: 1.03, 1.53).

**Table 2:** Multivariable-adjusted odds ratios and 95% confidence intervals associating patient demographic, tumor characteristics with receipt of chemotherapy, and racial disparities in those associations among breast cancer patients with discretionary indication for chemotherapy in Georgia (2010–2017)

	Discretionary indication for chemotherapy				
Patient demographic and tumor characteristics	(N)	Overall OR (95% CI)^a^	Treatment (N)		Stratified OR (95% CI)^a^
	Overall		NHW	NHB	
Overall disparity	2,872	**–**	1930	942	1.41 (1.28, 1.56)
Age at diagnosis					
> 70	182	0.72 (0.52, 0.98)	143	39	1.32 (0.92, 1.61)
55–70	1180	1.28 (1.08, 1.51)	845	335	1.33 (1.15, 1.55)
≤ 55	1510	Ref	942	568	1.51 (1.30, 1.75)
Stage					
I	1717	Ref	1,202	515	1.30 (1.14, 1.48)
II	1155	3.27 (2.95, 3.63)	728	427	1.38 (1.15, 1.66)
Tumor grade					
1	286	Ref	225	61	1.00 (0.74, 1.37)
2	1145	3.16 (2.74, 3.65)	836	309	1.04 (0.89, 1.23)
3 +	1395	15 (3, 18)	837	558	1.15 (0.96, 1.40)
ODX testing					
Testing not performed	2228	0.77 (0.69, 0.87)	1448	780	1.47 (1.31, 1.65)
Scored	644	Ref	482	162	0.90 (0.71, 1.14)
Marital status					
Single	1041	1.00 (0.91, 1.10)	543	498	1.37 (1.18, 1.60)
Married	1722	Ref	1324	398	1.50 (1.29, 1.74)
Socioeconomic Index					
0% – < 5% poverty	392	Ref	324	68	2.48 (1.70, 3.61)
5% – < 10% poverty	581	0.99 (0.84, 1.16)	447	134	1.46 (1.13, 1.88)
10% – < 20% poverty	1037	1.26 (1.09, 1.47)	681	356	1.40 (1.18, 1.67)
20–100% poverty	862	1.35 (1.15, 1.58)	478	384	1.16 (0.97, 1.39)
Urban/rural residence					
Urban	2382	Ref	1642	988	1.32 (0.91, 1.23)
Rural	489	0.95 (0.83, 1.08)	444	106	1.01 (0.76, 1.36)
Insurance type					
Uninsured	40	0.97 (0.64, 1.47)	22	22	1.12 (0.48, 2.63)
Private	1896	Ref	1392	683	1.44 (1.25, 1.65)
Medicaid	238	1.25 (1.03, 1.53)	129	156	1.15 (0.80, 1.66)
Medicare	584	0.80 (0.70, 0.92)	472	181	1.33 (1.08, 1.64)
Military	71	1.01 (0.74, 1.37)	39	42	1.37 (0.73, 2.55)

^a^Adjusted for: age, stage, grade, insurance status, poverty level, receipt of Oncotype DX, marital status, urban/rural

Table 2 also shows estimates for racial disparities (NHB vs NHW) in receipt of chemotherapy within strata of patient characteristics. Overall, we observed a racial disparity in chemotherapy initiation in our study—NHB women had 1.41-times to the odds of receiving chemotherapy (95% CI: 1.28, 1.56) than NHW women. However, the magnitude of the disparity varied across patient demographic and clinical characteristics. Among breast cancer patients who were ≤ 55 years at diagnosis, NHB women had 1.51 times the odds of receiving chemotherapy compared with NHW women (95% CI: 1.30, 1.75), whereas among women > 70 at diagnosis the disparity estimate was attenuated (OR = 1.32, 95% CI: 0.92, 1.61). Among breast cancer patients diagnosed with grade 1 tumors we did not observe a difference in receipt of chemotherapy between NHB and NHW women (OR = 1.00, 95% CI: 0.74, 1.37); however, with an increase in the tumor grade, NHB women had higher odds of receiving chemotherapy compared with NHW women. NHB women who did not receive Oncotype DX testing had 1.47 times the odds of receiving chemotherapy than NHW women (OR = 1.47, 95% CI: 1.31, 1.65); however, NHB women who received Oncotype DX testing had slightly lower odds of receiving chemotherapy than NHW women (OR = 0.90, 95% CI: 0.71, 1.14). The most pronounced disparity was observed among women residing in neighborhoods with the lowest proportion of residents living below the poverty index (OR = 2.48, 95% CI: 1.70, 3.61). Similarly, NHB women residing in urban settings had 1.32 times the odds of initiating chemotherapy than NHW women (OR = 1.32, 95% CI: 0.91, 1.23), whereas we did not observe a disparity among those residing in rural areas (OR = 1.01, 95% CI: 0.76, 1.36).NHB women with private insurance or Medicare had higher odds of initiating chemotherapy compared with NHW women (OR = 1.44, 95% CI: 1.25, 1.65 and OR = 1.33, 95% CI: 1.08, 1.64, respectively).

We additionally stratified the discretionary cohort by age at diagnosis (≥ 55 years vs. < 55 years) to investigate if the racial disparity we observed in the initiation of chemotherapy was driven by the on average younger age of diagnosis on average among NHB women (Table 3). We generally observed consistent estimates of association across the sociodemographic and clinical factors, as well as in the estimates on racial disparities in chemotherapy initiation. One exception was in tumor grade. Among those diagnosed < 55 years of age, NHB women had higher odds of initiating chemotherapy than NHW women among those diagnosed with grade 1 (OR = 1.53, 95% CI: 0.99, 2.35). On the other hand, among those diagnosed at 55 years of age or older, NHB women had lower odds of initiating chemotherapy among those diagnosed with grade 1 disease (OR = 0.61, 95% CI: 0.37, 0.99) than NHW women.

**Table 3:** Multivariable-adjusted odds ratios and 95% confidence intervals associating patient demographic, tumor characteristics with receipt of chemotherapy, and stratified by race and age among breast cancer patients with discretionary indication for chemotherapy in Georgia (2010–2017)

	Discretionary indication for chemotherapy (< 55 years of age)					Discretionary indication for chemotherapy (≥ 55 years of age)				
Patient demographic and tumor characteristics	(N)	Overall OR (95% CI)^a^	Treatment (N, %)		Stratified Effects OR (95% CI)^a^	(N)	Overall OR (95% CI)^a^	Treatment (N, %)		Stratified Effects OR (95% CI)^a^
	Overall		NHW	NHB		Overall		NHW	NHB	
Overall disparity	1425		889	536	1.50 (1.28, 1.75)	1447		1041	406	1.34 (1.17, 1.54)
Age at diagnosis										
> 70						182	0.67 (0.53, 0.86)	143	39	1.26 (0.88, 1.79)
≤ 70						1265	Ref	898	367	1.46 (1.27, 1.67)
Stage										
I	818	Ref	531	287	1.40 (1.15, 1.70)	899	Ref	671	228	1.22 (1.03, 1.45)
II	607	3.20 (2.71, 3.77)	358	249	1.40 (1.04, 1.88)	548	3.50 (3.07, 4.00)	370	178	1.36 (1.07, 1.73)
Tumor grade										
1	129	Ref	88	41	1.53 (0.99, 2.35)	157	Ref	137	20	0.61 (0.37, 0.99)
2	548	2.89 (2.31, 3.61)	387	161	1.04 (0.75, 1.44)	597	2.95 (2.44, 3.56)	449	148	1.08 (0.85, 1.38)
3 +	723	13.9 (11.8, 17.9)	396	327	0.97 (0.76, 1.24)	672	12.1 (9.90, 14.8)	441	231	1.12 (0.91, 1.39)
ODX testing										
Testing not performed	1098	0.91 (0.76, 1.08)	652	446	1.58 (1.31, 1.90)	1130	0.71 (0.61, 0.82)	796	334	1.38 (1.18, 1.61)
Scored	327	Ref	237	90	0.93 (0.66, 1.31)	317	Ref	245	72	0.90 (0.65, 1.24)
Marital status										
Single	492	1.12 (0.96, 1.31)	218	274	1.51 (1.22, 1.88)	549	0.98 (0.86, 1.10)	325	224	1.29 (1.06, 1.57)
Married	875	Ref	642	233	1.39 (1.08, 1.79)	847	Ref	682	165	1.50 (1.22, 1.84)
Socioeconomic Index										
0% – < 5% poverty	206	Ref	168	38	2.50 (1.41, 2.09)	205	Ref	171	34	2.30 (1.40, 3.76)
5% – < 10% poverty	286	0.90 (0.71, 1.15)	209	77	1.27 (0.89, 1.81)	311	1.03 (0.84, 1.27)	249	62	1.80 (1.27, 2.56)
10% – < 20% poverty	512	1.29 (1.03, 1.61)	305	207	1.33 (1.02, 1.74)	591	1.22 (1.01, 1.49)	414	177	1.45 (1.15, 1.83)
20–100% poverty	421	1.56 (1.22, 2.01)	207	214	1.29 (0.96, 1.75)	479	1.21 (0.98, 1.49)	291	188	1.03 (0.82, 1.30)
Urban/rural residence										
Rural	219	0.97 (0.77, 1.21)	169	50	1.12 (0.68, 1.83)	270	0.95 (0.81, 1.12)	226	44	0.93 (0.64, 1.36)
Urban	1206	Ref	720	486	1.35 (1.13, 1.62)	1177	Ref	815	362	1.31 (1.12, 1.53)
Insurance type										
Uninsured	21	0.92 (0.49, 1.72)	9	12	2.42 (0.67, 8.69)	19	0.97 (0.56, 1.68)	14	5	0.57 (0.16, 2.02)
Private	1150	Ref	759	391	1.41 (0.76, 2.61)	746	Ref	544	202	1.48 (1.20, 1.83)
Medicaid	157	1.39 (1.05, 1.83)	68	89	1.12 (0.67, 1.87)	81	1.07 (0.80, 1.42)	40	41	1.19 (0.69, 2.04)
Medicare	36	0.65 (0.42, 1.01)	18	18	1.96 (0.83, 4.64)	548	0.99 (0.85, 1.17)	408	140	1.27 (1.02, 1.59)
Military	42	1.14 (0.72, 1.80)	22	20	0.79 (0.32, 1.94)	29	0.87 (0.58, 1.37)	15	14	2.18 (0.91, 5.21)

^a^Adjusted for: age, stage, grade, insurance status, poverty level, receipt of Oncotype DX, marital status, urban/rural status

In Table 4 we present the racial disparities in breast cancer mortality by receipt of chemotherapy. Among those with discretionary indication for chemotherapy and who received chemotherapy, NHB women had 1.31 times the estimated mortality rate compared with NHW women (95% CI: 0.95, 1.81). Among those who did not receive chemotherapy, the disparity estimate was similar (HR = 1.41, 95% CI: 1.04, 1.91).

**Table 4:** Hazard ratios (HR) and 95% confidence intervals (95% CI) associating chemotherapy receipt with breast cancer-specific mortality overall and by race among patients with discretionary indication for chemotherapy in Georgia (2010–2017)

	No. events		Racial disparity	
	NHW	NHB	HR (95%CI)^a^	HR (95%CI)^b^
Discretionary for chemotherapy				
Chemotherapy				
Yes	106	59	1.31 (0.95, 1.81)	1.08 (0.77, 1.52)
No	166	55	1.41 (1.04, 1.91)	1.19 (0.85, 1.67)

^a^Age-adjusted

^b^Adjusted for: age, stage, grade, insurance status, poverty level, node status, receipt of Oncotype DX, marital status, urban/rural

*NHW* non-Hispanic White, *NHB* non-Hispanic Black

In our sensitivity analyses among ER-positive breast cancer patients with limited lymph node involvement we observed comparable results (Supplemental Tables 2, 3 and 4).

## Discussion

In this study, we investigated differences in the initiation of chemotherapy across patient demographic and clinical characteristics among breast cancer patients diagnosed with ER-positive tumors who were considered to have a discretionary indication for chemotherapy per NCCN guidelines. We observed that NHB women were more likely, overall, to initiate chemotherapy than their NHW counterparts. However, this disparity was particularly pronounced among women diagnosed 70 years or younger, with higher stage disease, patients who did not receive Oncotype DX testing, women living in high SES neighborhoods, and those with private insurance at the time of diagnosis. We did not observe racial disparities in the initiation of chemotherapy among women who received Oncotype DX testing, were diagnosed with grade 1 tumors, or among breast cancer patients residing in rural areas.

Few studies have investigated sociodemographic factors that impact chemotherapy initiation among patients with a discretionary chemotherapy indication [14, 23, 24]. Consistent with prior studies, we observed that patients diagnosed at age 70 years or younger were more likely to initiate chemotherapy than those diagnosed over the age of 70. However, trials have indicated that age alone is insufficient to inform decisions regarding the initiation of chemotherapy [25]. In the current study, we did not have information regarding comorbid conditions present at diagnosis, which also influence the decision to initiate chemotherapy, especially among older breast cancer patients.

In our study, NHB women were more likely to initiate chemotherapy across nearly all patient demographic and clinical characteristics. This may indicate that NHB patients are being systematically over-treated with adjuvant chemotherapy when the indication is discretionary. We did not observe racial disparities in the initiation of chemotherapy among those who received Oncotype DX testing, suggesting that in the absence of following all clinical guidelines, NHB breast cancer patients are more likely to be over-treated with chemotherapy. Oncotype DX testing has led to biomarker-driven treatment among breast cancer patients that may contribute to more equitable care decisions in this group. We also did not observe racial disparities among those who resided in rural settings. The lack of disparities among those residing in rural settings may be driven by barriers in access to care for both NHB and NHW breast cancer patients, or due to providers being able to treat patients more equally as the patient volume is lower and there are more opportunities to bring patients to cancer centers [26]. Our results support the idea that when clinical guidelines are followed, patients receive the same recommended treatments, and overtreatment can be avoided.

We observed that NHB women had higher breast cancer mortality rates than NHW women, regardless of chemotherapy receipt. This may suggest that despite more aggressive treatment with chemotherapy among those with discretionary treatment indication, NHB women are still more likely to die from their disease. However, our estimates were imprecise due to a limited number of deaths due to breast cancer and additional studies with longer follow-up are needed. Further exploration of factors contributing to the differences in breast cancer mortality between Black and White women diagnosed with prognostically favorable tumors is necessary.

There are limitations to this study worth noting. First, we did not have information on functional status or comorbidities present at diagnosis, which would influence the decision-making process on whether to initiate chemotherapy. The risk of chemotherapy-related toxicities is higher among patients with low functional status or a high comorbidity burden, which would affect the oncologist’s willingness to proceed with chemotherapy among those with a discretionary indication. However, we expect that the comorbidity burden would be higher among NHB patients, and we did not observe lower chemotherapy initiation among NHB patients. Second, the time period of breast cancer diagnoses included in this cohort used Oncotype DX testing results prior to the results presented from the TAILORx and RxPONDER trials, which now guide chemotherapy guidelines [27]. Third, we did not consider tumor histology due to the low proportion in our sample with unfavorable tumor histologies. Future studies may want to consider tumor histology as well as breast cancer subtype as factors that influence chemotherapy receipt. Fourth, chemotherapy is often under ascertained by cancer registries and may suffer from misclassification. Finally, as this was a population-based registry study, we were unable to assess the decisions by the patient and physician to initiate chemotherapy, which would be informative for future work. Future studies would likely benefit from information regarding the decision-making process from patient and physician perspectives.

In this study, we observed differences by sociodemographic factors in the initiation of chemotherapy among breast cancer patients with a discretionary indication for chemotherapy. We also observed racial disparities in the initiation of chemotherapy overall and by sociodemographic factors. Overtreatment of chemotherapy may lead to an increase in adverse outcomes among breast cancer patients. Therefore, adherence to clinical guidelines can lead to equitable treatment among breast cancer patients. Further research may be warranted to understand potential adverse outcomes among NHB breast cancer patients who initiate chemotherapy when care is discretionary.

### Supplementary Information

Below is the link to the electronic supplementary material.

Supplementary file1 (DOCX 30 KB)

## Data Availability

The data are publicly available from the Georgia Cancer Registry, a cancer registry that is part of the Surveillance, Epidemiology, and End Results (SEER) program that provides information on cancer statistics in the United States.
