# Supplementary material for: Racial disparities in initiation of chemotherapy among breast cancer patients with discretionary treatment indication in the state of Georgia
Source: Breast Cancer Res Treat. 2024 Mar 22;205(3):609–18. doi: 10.1007/s10549-024-07279-w (PMC11101533; doi:10.1007/s10549-024-07279-w)
Supplement: Supplementary file 1 — Supplementary file1 (DOCX 30 KB) [file 10549_2024_7279_MOESM1_ESM.docx]

| **Table S1: Summary of adjuvant chemotherapy use for estrogen receptor positive breast cancer patients based on the 2016 National Comprehensive Cancer Network guidelines** | | | | |  |
| --- | --- | --- | --- | --- | --- |
| **Tumor Subtype** | **Lymph Node Status** | **Tumor Size (cm)** | **Oncotype DX recurrence score** | **Chemotherapy Recommendation** | |
| ER+/HER2+ | Negative | ≤1.0 | NA | Discretionary | |
|  |  | >1.0 | NA | Yes | |
|  | Positive | All | NA | Yes | |
| ER+/HER2- | Negative | ≤0.5 | NA | No | |
|  |  | >0.5-5cm | Low (<18) | No | |
|  |  |  | Intermediate (18-30) | Discretionary | |
|  |  |  | High (>30) | Yes | |
|  |  |  | Not done | Discretionary | |
|  | Positive | All | NA | Yes | |

| **Table S2**: Demographic, tumor, and patient characteristics by receipt of chemotherapy among non-Hispanic Black (NHB) and non-Hispanic White (NHW) women diagnosed with ER+ breast cancer and limited lymph node involvement in Georgia (2010-2017). | | | | |
| --- | --- | --- | --- | --- |
|  | **Chemotherapy Initiation** | | | |
|  | **Yes** | | **No** | |
|  | **(n=3,174)** | | **(n=1926)** | |
|  | **Median** | **IQR** | **Median** | **IQR** |
| **Age at Diagnosis (years)** | 55 | 46, 63 | 66 | 56, 75 |
| **Length of Follow-up (months)** | 68 | 45, 93 | 58 | 39, 85 |
|  | **N** | **%** | **N** | **%** |
| **Breast Cancer-Specific Death** | 274 | 5.4 | 129 | 2.5 |
| **Race and Ethnicity** |  |  |  |  |
| NHB | 1064 | 71 | 438 | 29 |
| NHW | 2110 | 58 | 1488 | 41 |
| **Age at Diagnosis** |  |  |  |  |
| >70 | 233 | 25 | 690 | 75 |
| ≤70 | 2941 | 70 | 1236 | 30 |
| **Stage** |  |  |  |  |
| I | 230 | 33 | 473 | 67 |
| II | 2508 | 65 | 1358 | 35 |
| III | 435 | 82 | 95 | 18 |
| **Tumor Grade** |  |  |  |  |
| 1 | 451 | 44 | 580 | 56 |
| 2 | 1574 | 60 | 1058 | 40 |
| 3+ | 1063 | 80 | 262 | 20 |
| Unknown | 86 | 77 | 26 | 23 |
| **Tumor size (cm)** |  |  |  |  |
| ≤0.5 | 67 | 59 | 47 | 41 |
| 0.6–1 | 226 | 52 | 211 | 48 |
| >1 to <5 | 2478 | 61 | 1577 | 39 |
| ≥5 | 385 | 81 | 89 | 19 |
| **Lymph Node Positivity** |  |  |  |  |
| 1 | 1829 | 55 | 1481 | 45 |
| 2 | 856 | 71 | 347 | 28 |
| 3 | 489 | 83 | 98 | 17 |
| **ODX testing** |  |  |  |  |
| Not performed | 2813 | 69 | 1237 | 31 |
| High | 58 | 85 | 10 | 15 |
| Medium | 184 | 52 | 171 | 48 |
| Low | 119 | 19 | 508 | 81 |
| **Demographic Characteristics** | | | | |
| **Marital Status** |  |  |  |  |
| Single | 501 | 70 | 217 | 30 |
| Married (common law and unmarried domestic) | 1841 | 65 | 986 | 35 |
| Other (divorced, widowed, separated | 696 | 52 | 640 | 48 |
| Unknown | 136 | 62 | 83 | 38 |
| **Socioeconomic Index** |  |  |  |  |
| 0% – <5% poverty | 407 | 58 | 297 | 42 |
| 5% – <10% poverty | 641 | 62 | 401 | 38 |
| 10% – <20% poverty | 1097 | 63 | 652 | 37 |
| 20% – 100% poverty | 1029 | 64 | 576 | 36 |
| **Urban/rural residence** |  |  |  |  |
| Urban | 2611 | 63 | 1560 | 37 |
| Rural | 563 | 61 | 366 | 39 |
| **Insurance Type** |  |  |  |  |
| Uninsured | 73 | 76 | 23 | 24 |
| Private | 1988 | 71 | 803 | 29 |
| Medicaid | 327 | 77 | 98 | 23 |
| Medicare | 681 | 42 | 943 | 58 |
| Military | 72 | 71 | 29 | 29 |
| Unknown | 31 | 51 | 30 | 49 |

| **Table S3.** Multivariable-adjusted odds ratios and 95% confidence intervals associating patient demographic, tumor characteristics with receipt of chemotherapy, and racial disparities in those associations among ER-positive breast cancer patients with limited lymph node involvement in Georgia (2010-2017)**.** | | | | | |
| --- | --- | --- | --- | --- | --- |
|  | **Chemotherapy indication, LN 1-3** | | | | |
| **Patient demographic and tumor characteristics** | **(N)** | **Overall  OR (95% CI)^1^** | **Treatment (N)** | | **Stratified  OR (95% CI)^1^** |
|  | Overall |  | NHW | NHB |  |
| **Overall Disparity** | 3174 | **--** | 2110 | 1064 | 1.39 (1.21, 1.61) |
| **Age at Diagnosis** |  |  |  |  |  |
| >70 | 233 | 0.50 (0.40, 0.62) | 172 | 61 | 1.55 (1.09, 2.20) |
| ≤70 | 2941 | Ref. | 1938 | 1003 | 1.37 (1.18, 1.60) |
| **Stage** |  |  |  |  |  |
| I | 230 | Ref. | 154 | 76 | 1.75 (1.18, 2.60) |
| II | 2508 | 4.94 (4.08, 5.98) | 1693 | 815 | 1.24 (1.05, 1.47) |
| III | 435 | 14 (10, 19) | 263 | 172 | 1.01 (0.60, 1.70) |
| **Tumor Grade** |  |  |  |  |  |
| 1 | 451 | Ref. | 329 | 122 | 1.52 (1.11, 2.10) |
| 2 | 1574 | 1.99 (1.70, 2.33) | 1095 | 479 | 1.24 (1.01, 1.51) |
| 3+ | 1063 | 5.21 (4.27, 6.36) | 633 | 430 | 1.06 (0.78, 1.46) |
| **ODX testing** |  |  |  |  |  |
| Testing not performed | 2813 | 4.50 (3.81, 5.31) | 1857 | 956 | 1.16 (0.98, 1.38) |
| Scored | 361 | Ref. | 253 | 108 | 1.50 (1.08, 2.09) |
| **Marital Status** |  |  |  |  |  |
| Single | 1191 | 0.97 (0.85, 1.11) | 618 | 573 | 1.62 (1.32, 1.99) |
| Married | 1847 | Ref. | 1412 | 435 | 1.23 (0.99, 1.52) |
| **Socioeconomic Index** |  |  |  |  |  |
| 0% – <5% poverty | 407 | Ref. | 341 | 66 | 1.81 (1.06, 3.11) |
| 5% – <10% poverty | 641 | 1.22 (0.98, 1.52) | 498 | 143 | 1.41 (0.97, 2.06) |
| 10% – <20% poverty | 1097 | 1.40 (1.14, 1.71) | 747 | 350 | 1.43 (1.11, 1.85) |
| 20% – 100% poverty | 1029 | 1.54 (1.24, 1.90) | 524 | 505 | 1.21 (0.95, 1.54) |
| **Urban/rural residence** |  |  |  |  |  |
| Urban | 563 | Ref. | 431 | 132 | 1.28 (1.08, 1.51) |
| Rural | 2611 | 1.10 (0.92, 1.31) | 1679 | 932 | 1.07 (0.73, 1.57) |
| **Insurance Type** |  |  |  |  |  |
| Uninsured | 73 | 1.17 (0.70, 1.95) | 39 | 34 | 0.88 (0.32, 2.39) |
| Private | 1988 | Ref. | 1361 | 627 | 1.50 (1.22, 1.86) |
| Medicaid | 327 | 1.24 (0.95, 1.62) | 147 | 180 | 1.09 (0.67, 1.78) |
| Medicare | 681 | 0.93 (0.78, 1.11) | 488 | 193 | 1.30 (1.01, 1.68) |
| Military | 72 | 1.20 (0.75, 1.94) | 49 | 23 | 0.89 (0.32, 2.47) |
|  | ^1^Adjusted for: age, stage, grade, number of positive nodes, insurance status, poverty level, node status, receipt of Oncotype DX, marital status, urban/rural | | | |  |
|  |  |  |  |  |  |

| **Table S4.** Hazard ratios (HR) and 95% confidence intervals (95% CI) associating chemotherapy receipt with breast cancer-specific mortality overall and by race among ER-positive breast cancer patients with limited lymph node involvement in Georgia (2010-2017) | | | | |  |
| --- | --- | --- | --- | --- | --- |
|  | **No. events** | | **Racial Disparity** | |  |
|  | **NHW** | **NHB** | **HR (95%CI)^1^** | **HR (95%CI)^2^** |  |
|  |  |  |  |  |  |
| Chemotherapy |  |  |  |  |  |
| Yes | 159 | 115 | 1.51 (0.88, 1.92) | 1.14 (0.87, 1.48) |  |
| No | 93 | 36 | 1.47 (1.00, 2.16) | 1.08 (0.72, 1.62) |  |
| ^1^Age-adjusted | | | | |  |
|  |  |  |  |  |  |
| ^2^Adjusted for: age, stage, grade, insurance status, poverty level, node status, receipt of Oncotype DX, marital status, urban/rural | | | | |  |
|  |  |  |  |  |  |
